# Proteolysis dysfunction in the process of aging and age-related diseases

**DOI:** 10.3389/fragi.2022.927630

**Published:** 2022-07-22

**Authors:** Natalia Frankowska, Katarzyna Lisowska, Jacek M. Witkowski

**Affiliations:** Department of Physiopathology, Faculty of Medicine, Medical University of Gdansk, Gdańsk, Poland

**Keywords:** aging, proteostasis, chaperones, ubiquitin-proteasome system, autophagy, lysosome, proteases

## Abstract

In this review, we discuss in detail the most relevant proteolytic systems that together with chaperones contribute to creating the proteostasis network that is kept in dynamic balance to maintain overall functionality of cellular proteomes. Data accumulated over decades demonstrate that the effectiveness of elements of the proteostasis network declines with age. In this scenario, failure to degrade misfolded or faulty proteins increases the risk of protein aggregation, chronic inflammation, and the development of age-related diseases. This is especially important in the context of aging-related modification of functions of the immune system.

## Highlights

• Protein quality control systems (proteostasis) are responsible for the functionality and turnover of cellular proteins.
• During aging, the cellular environment is more prone to damage due to progressing failure in maintaining cellular protein homeostasis, which is related to the increased frequency of chronic age-related diseases.
• The composition of the proteome of the cells of the elderly differs from young individuals.

## 1 Introduction

For an eucaryotic organism to function correctly, it needs to respond effectively to external and internal factors. The effectors of these responses are mainly thousands of different intra- and extracellular proteins. During aging, there is a noticeable, progressive decline in the functionality of an organism on many different levels, including notably the cellular, subcellular and molecular, making it more vulnerable, prone to diseases, and eventually leading to its death (Ruano, 2021). This decline is associated with changes in the cellular proteome, being the consequence of adverse effects of aging for every stage of protein manufacturing and maturation. More and more damaged or malformed proteins accumulate over time, causing overall cell deterioration (Hipp et al., 2019). Thus, the dynamic balance between the production of functional proteins (including their translation, posttranslational modifications, multi-order folding leading to functional proteome), maintenance of the proteome and detection/degradation of dysfunctional, misfolded, or aggregated proteins is failing with time. Altogether the mentioned processes of protein synthesis, folding and maintenance, and finally degradation are described as proteostasis network (PN) or proteodynamics (Balch et al., 2008; Hipp et al., 2019; Jayaraj et al., 2020; Witkowski et al., 2021). One needs to bear in mind that this cellular protein homeostasis machinery is also a network of proteins performing quality control of other cellular proteins and, as such, is prone to similar aging-related changes.

There are nine distinguishable hallmarks of aging that include cellular and molecular changes. They are divided into the following categories: genomic instability, telomere attrition, epigenetic alterations, deregulated nutrient sensing, mitochondrial dysfunction, cellular senescence, stem cell exhaustion, altered intercellular communication, and last but not least, loss of proteostasis (López-Otín et al., 2013). Impaired proteostasis can lead to failure in protein refolding and degrading, promoting proteotoxicity. Eventually, such a scenario causes an imbalance in a dynamic state of proteome and interferes with the manufacturing of functionally active proteins, leading to cellular dysfunction and death. In a broader perspective, these changes that take place on molecular and cellular level influence maintaining of body homeostasis and cause impairment in physiological systems, including the immune system (de Toda et al., 2021). Impaired proteostasis network is notably included among the hallmarks of aging of specific types of cells characterized by altogether halted proliferation like the neurons, heart muscle, and striated muscle fibers (Wiersma et al., 2016) or by periods without proliferative activity, like the T and B lymphocytes and macrophages (Mittelbrunn and Kroemer, 2021; Zhou et al., 2021).

The cellular environment is constantly exposed to various stress stimuli, not to mention cellular metabolism associated with the production of free radicals and oxidants. In the concept of the free radical theory of aging (Harman, 1956), oxidation is proposed to be the main damaging modification of proteins and other biomolecules during aging. Exposure of the cells to reactive oxygen species (ROS) results in elevated amounts of oxidized proteins and an increased risk for protein aggregation at the same time. Also, other aging-related processes, including accumulation of SNPs and somatic mutations, decreased fidelity of transcription and translation, and changes in posttranslational protein modification will add to the rising proportion of misfolded and aggregated proteins in aging cells (Witkowski et al., 2021). Irreversible accumulation of modified (e.g., misfolded, oxidized, or improperly cleaved) proteins contributes to the development of aging-related diseases that include neurodegenerative disorders such as Parkinson’s disease or Alzheimer’s disease (Ho et al., 2020; Pathak et al., 2021), metabolic disorders (Sooparb et al., 2004; Kimura et al., 2015) or even cancer (Fulop et al., 2013; Chavez-Dominguez et al., 2020).

## 2 Components contributing to proteostasis

### 2.1 Molecular chaperones

In most cases, newly-made proteins (when their amino acid sequence possesses information for folding) must assemble in three-dimensional structures, which is a conformation that is required for their biological activity and functionality. In a cellular environment, many kinds of distinct proteins reside together in high concentrations in little space, interact and affect each other (Hartl et al., 2011). In order to counteract misfolding and aggregation of nascent proteins, a set of different molecular chaperones operates within cells. The molecular chaperone is defined as any protein that can either interact with, stabilize or help the other protein to obtain its functional conformation and is not present in its final structure. Continual surveillance by chaperones and their presence in all cellular compartments enables efficient protein folding, consequently preventing aggregation.

Molecular chaperones are divided into families of different cooperating types forming five major classes (HSP60s, HSP70s, HSP90s, HSP104s, and small HSPs). Besides assisting with folding newly synthesized proteins, they are also involved in refolding stress-modified proteins, oligomeric assembling, or even protein trafficking (Saibil, 2013). Commonly known family members of molecular chaperones are so-called heat-shock proteins (HSPs) and are mainly upregulated in response to stress factors where the heat shock transcription factors (HSFs) act as direct transcriptional inducers of genes that encode molecular chaperones. The elevated expression level of HSPs can be driven by HSFs for instance in a situation caused mainly due to the increasing number of misfolded or partially folded protein intermediates that are prone to aggregation in the cellular environment (Hartl et al., 2011). It has also been proven that HSPs can be constitutively expressed during development and differentiation without stressful stimuli. During the myogenesis process, the myogenic transcription factor HLH-1 (MyoD) regulates the expression of chaperone genes and therefore folds muscle proteins (Bar-Lavan et al., 2016). The mechanism of protein folding (at larger chaperones of a molecular mass between 40 and 105 kDa) is mainly based on repeated cycles of ATP binding and hydrolysis, where hydrophobic amino acid fragments are exposed in polypeptide chains. There are also small, ATP-independent HSPs (of a molecular mass between 8 and 28 kDa) that function as so-called holdases that buffer aggregation (Miller and Fort, 2018). Chaperones are characterized by relatively low substrate specificity and work on a wide range of proteins (Saibil, 2013).

The chaperones that have the most substantial contribution to maintaining proteome functionality are cytosolic HSP90s, HSP70s, and HSP60s (chaperonins) and small HSPs as well. Members of the HSP70 family have been described as having the most prominent role in response to stress stimuli, particularly high temperatures (Koga et al., 2011). The HSP70 system is also constitutively expressed, and its effectiveness was proven in a *Drosophila* model for Parkinson’s disease by preventing the formation of toxic protein aggregates and therefore warding off a dopaminergic neuronal loss (Auluck et al., 2002). The HSP90 forms are involved in several signaling pathways in eukaryotic cells, including apoptosis, vesicle-mediated transport, or targeted protein degradation (Hartl et al., 2011). The chaperonins form double-ring complexes that act as containers enabling the folding of other protein subunits. HSP60s were extensively studied in the *E. coli* system. In eukaryotic cells, they interact with a minority of newly synthesized proteins in the cytosol, such as actin or tubulins. They are also useful in preventing the aggregation of toxic protein products in Huntington’s disease (Ranson et al., 1998; Tam et al., 2006). Small HSPs take part in diverse cellular processes and play a role in stress tolerance. Except preventing proteins from aggregation, they are able to suppress production of reactive oxygen species and posses immunomodulatory and anti-inflammatory properties as well (Bakthisaran et al., 2015).

Distinct, organelle-specific chaperones are dedicated to controlling the proper folding of proteins located in the membrane and lumen of various organelles inside cells. Here, the endoplasmic reticulum (ER) chaperones are the ones that are the best characterized. When impaired protein folding is detected in ER lumen, they participate in the so-called unfolded protein response (UPR) that eventually passes information of its status to the nucleus and cytosol to enhance the cell’s protein-folding capacity. Protein folding balance is restored *via* the UPR action, for example, by decreasing the number of proteins that must be folded or eliminating the part of the unfolded proteins which last longer to fold. When proper balance is restored, the UPR is inactivated; if not, it can result in cell death (Read and Schröder, 2021).

The role of the chaperone system for the efficient function of the immune system cells is indispensable. It has a special role in the maturation of proteins of the major histocompatibility class I (MHC I), starting from their synthesis, through maturation and folding, intracellular trafficking *via* the secretory pathway, antigenic cargo optimization, release of the ready molecule to the cell surface, and finally in binding with their cognate receptors on cytotoxic CD8^+^ T cells (Thomas and Tampé, 2019; Trowitzsch and Tampé, 2020). Specialized chaperones are also involved in the adequate maturation and transport of MHC II molecules in the antigen-presenting cells (APCs) (Dijkstra and Yamaguchi, 2019; Sadegh-Nasseri and Kim, 2019).

### 2.2 Main intracellular proteolytic systems

The protein quality control systems tightly control the composition of the proteome in the cellular environment. Proteins that are misfolded or malfunction must undergo a degradation process in spatially controlled cell compartments. The main intracellular proteolytic systems responsible for protein turnover comprise the proteasome/immunoproteasome and the lysosome. Apart from that, there is another group of “free” cytoplasmic proteases that includes calpains, caspases, and a desintegrase and metalloproteinase (ADAM) family. These, rather than or in addition to function in protein quality control, perform multiple protein-modifying actions, thus serving as switches of these proteins’ functionalities. Most of these protein quality control systems will be discussed in this article.

#### 2.2.1 Ubiquitin-proteasome system

An ATP-dependent degradation of 80% of cellular proteins is catalyzed by a macromolecular complex called the 26S proteasome that preferentially degrades these proteins that are soluble and poly-ubiquitinated (Hershko and Ciechanover, 1986). The 26S proteasome is composed of the 20S complex having the proteolytic core and the 19S, which is a regulatory complex that consists of heat-shock proteins, ATPases, and enzymes removing ubiquitin tags from the substrate proteins. The 19S proteasome thus provides the connection between the proteasome-mediated proteolysis and the ubiquitination process (Navon and Ciechanover, 2009). In high eukaryotes, the 20S proteasome is built of 4 rings (α-subunits form the outer rings and β-subunits the inner rings, respectively), with each one containing seven separate subunits forming together a cylinder-shaped structure (Hershko and Ciechanover, 1992).

The 20S proteasome is made up of threonine proteases that exhibit caspase-, trypsin-, and chymotrypsin-like activities (Finley et al., 2016); in effect, the product of proteasomal degradation is typically a short peptide, consisting of 8-12 residues. Interestingly, such peptides can be incorporated into other processes that include antigen presentation by the major histocompatibility complex (MHC) class I pathway (Galluzzi et al., 2017).

That proteasome complex is responsible for an enzymatic breakdown of oxidized, damaged, or misfolded proteins. It is also involved in the physiological and biochemical intracellular processes, including cell growth and differentiation, DNA replication and repair, or even cell metabolism and immune system response (Wang and Le, 2019).

For protein degradation by the 26S proteasome, the protein needs to be initially tagged with ubiquitin, a conserved 76 amino acids long protein with seven lysine residues in its chain. In the ubiquitination process, a covalent linkage of the ubiquitin is conducted by a group of enzymes in the following steps. The process begins with the activation of the ubiquitin by the E1 enzyme. The molecule that has been activated is then transferred to the member of the family of E2 enzymes, which play the role of ubiquitin-conjugating enzymes. In a final step, one of the widely distributed E3 ligases will bind to a ubiquitin-carrying E2 enzyme and catalyze the conjugation of ubiquitin to the lysine residues of the target protein. Cycles of ubiquitination can be repeated, resulting in manufacturing the polyubiquitin chain, which is further recognized by the elements of regulatory complex 19S of the proteasome (Ciechanover, 1993). Different levels of ubiquitination, starting from addition of just one molecule of ubiquitin to the target protein, as well as different lysine (K) residues of ubiquitin used for its conjugation with the substrate protein, allow for more precise control of the protein fate. For instance, mono-ubiquitination serves as signal for the regulation of DNA repair or gene expression. Polyubiquitination through K48 of ubiquitin leads to proteasomal degradation, and K63-linked ubiquitin plays a role in signaling, endocytosis, or even regulating kinase activation (Sadowski and Sarcevic, 2010). It was demonstrated that ubiquitination *via* K63 is important for the regulation of signal transduction by multiple receptors of both innate and adaptive immunity, including the TLRs, NLRs, TNFR, T- and B-cell antigen receptors and many other (Madiraju et al., 2022).

##### 2.2.1.1 Immunoproteasome

It has been demonstrated that interferon-gamma (IFN-γ) and tumor necrosis factor-alpha (TNF-α) cause the replacement of the constitutive catalytic subunits (β1, β2, and β5) of the proteasome with the inducible catalytic subunits (β1i, β2i, and β5i), which results in forming of so-called immunoproteasome that additionally interacts with the regulatory proteasome activator complex PA28 (11S) and is more efficient in performing proteolytic functions (Gavilán et al., 2012). A fundamental role of the immunoproteasome is processing antigens for presentation on MHC class I molecules to cytotoxic T cells (CD8^+^). Thus, the immunoproteasome has a prominent role in activating the immune system against cells infected by viruses or neoplastically transformed (Kimura et al., 2015). Recent findings also suggest that immunoproteasome induction is essential for the immune response and the preservation of proteostasis under stress situations. Thus, apart from participating in antigen presentation (Strehl et al., 2005), the immunoproteasome is (in addition to UPS and autophagy) capable of targeting for degradation of nascent oxidant-damaged proteins (DRiPs) that were the result of interferon-induced oxidative stress (Seifert et al., 2010).

#### 2.2.2 Autophagy-lysosome pathway

We can distinguish three ways of possible autophagic degradation of cytoplasmic material of exogenous or endogenous origin. Nevertheless, these autophagic responses are dependable on lysosomal activity but differ in the molecular mechanisms they operate on (Galluzzi et al., 2017). In microautophagy, cytoplasmic cargo is directly taken up by the lysosome. Macroautophagy is associated with morphological changes in vesicular compartments with the ability to ingest a sizeable part of the cytoplasm. The last possible mechanism concerns chaperone-mediated autophagy (CMA), where cytosolic proteins destined for degradation are delivered to the lysosomal lumen.

Microautophagy relies on the direct capture of cytosolic material through a lysosomal membrane invagination. It is still a poorly characterized mechanism in mammalian cells, unlike yeast or plants (Schuck, 2020). The lysosome, an organelle bound by a single lipid bilayer, supplies the cell with a variety of almost 60 hydrolases (including proteases, glycosidases, lipases, and nucleases). The highest activity of these enzymes occurs at low pH values of 4.5–5.5; the necessary highly acidic environment inside the lysosome is maintained by membrane-bound proton pumps (Mony et al., 2016). The significant difference between the ubiquitin-proteasome and the lysosomal systems is that the UPS degrades only proteins, whereas the latter can digest different macromolecules, aggregates, or even an extracellular material. The autophagy-lysosome system also plays a fundamental role in maintaining cellular homeostasis. Since the lysosomal hydrolases catabolize their substrates to the most elementary forms of compounds, there is an available recycled material for synthesizing biomolecules and performing metabolic reactions. This catabolic pathway, among others, initiates apoptosis, tissue remodeling, and cholesterol homeostasis (Yang and Klionsky, 2010).

Contrary to microautophagy, the autophagic process of macroautophagy has been investigated more thoroughly. In this case, the catabolic operation starts with incorporating cytosolic cargo by a *de-novo* generated vesicle with a continuous membrane called the phagophore. After that, the autophagosome formation is involved, a double-membrane vesicle that originated from the phagophore. Once the cytosolic cargo is sequestered, and the autophagosome is sealed, the content of the vesicle is intended for degradation *via* fusion with the lysosome, resulting in the formation of autolysosome (Pavel and Rubinsztein, 2017). The above-mentioned mechanism is typically induced by stress factors such as inefficient nutrients, malfunctioning mitochondria, protein aggregates, or pathogens. With the aim of autophagosome formation, a set of autophagy-related genes (ATG) is expressed, evolutionarily well-preserved from yeast to mammals. So-called core machinery includes approximately 20 ATG genes essential for most subtypes of autophagy (Ohsumi, 2006).

All the above-mentioned macroautophagy processes are present also in the cells of the adaptive immune system. Thus, macroautophagy is believed to be crucial for lymphocyte homeostasis, both for B and T cells, as well as for the survival of effector plasma cells. In all these cell types it seems to play an important role in glucose and lipid metabolism and in maintenance of functional mitochondria and endoplasmic reticulum. Finally, macroautophagy is implicated also in antigen presentation by B lymphocytes. Various autophagy-related gene (ATG) products, including beclin1; Atg 3, 5, 7, 12, 16L1; WIPI2, and LAMP2A are engaged in the development, activation, and apoptosis of either B or T or NK lymphocytes (Arbogast and Gros, 2018).

Many studies have shown that there are also signal transduction pathways involved in regulating autophagy. The mechanistic target of rapamycin (mTOR) belongs to one of the regulatory serine/threonine-protein kinases from the phosphoinositide 3 kinase-related kinase (PKK) family. It performs the function of central regulatory protein converging signals (nutrition level or energy resources) from inside and outside of the cellular environment, thereby managing cell growth, proliferation, and protein synthesis (Wang and Zhang, 2019). In that manner, the induction of the autophagy process is negatively regulated by mTOR. In the case of a cell being provided with nutrients, mTOR subsequently induces phosphorylation of proteins responsible for the autophagy induction step (Atg1 and Atg13). Such interaction finally results in the suppression of the autophagy process. As described in several publications (Harrison et al., 2009; Sutton et al., 2018; Green et al., 2022), in order to increase the life span and because improper nutrient sensing is another of the nine pillars of cellular aging, macroautophagy is activated with the use of mTOR inhibitors or caloric restriction. A commonly used inhibitor is rapamycin, a pharmacological agent proven to effectively block the growth of eukaryotic cells (Selvarani et al., 2021).

Chaperone-mediated autophagy classifies as the third type of autophagy. As opposed to micro- and macroautophagy, it is targeted against single proteins removed selectively. The recognition of unfolded protein can be executed by the cytosolic heat-shock cognate chaperone of 70 kDa (Hsc70) (Kaushik and Cuervo, 2018). Owing to the presence of a unique KFERQ motif in the sequence of cytosolic substrate protein, hsc70 specifically binds to the substrate and forms a chaperone-substrate complex along with other heat-shock proteins (such as Hsp90 or Hsp40). This complex can bind then to lysosome-associated membrane protein type 2A (LAMP2A), and the assistance of a chaperone allows for unfolding the substrate protein. Subsequently, LAMP2A undergoes multimerization and enables direct translocation of the targeted protein across the lysosomal membrane (Juste and Cuervo, 2019). The most noticeable activity of CMA is noted under the conditions that result in protein damage or when the cellular environment faces oxidative stress or deficiency of nutrients. Thus selectivity of CMA enables the degradation of only modified molecules without disturbing the protein balance nearby.

#### 2.2.3 Calpain system

Calpains comprise the evolutionarily conserved family of neutral cytosolic cysteine proteases (Sorimachi et al., 2011). Their proteolytic activity strictly depends on a high concentration of calcium ions that greatly exceed a normal resting concentration of Ca^2+^ in the cytosol of resting cells, estimated as around 100 nM. The two first experimentally documented calpains (out of 14 described so far) are called conventional due to their ubiquitousness, similarity in sequence, and similar time of discovery (Yoshimura et al., 1983). In nomenclature, they come with the names: calpain I (μ-calpain) and calpain II (m-calpain) and are recognized by the concentration of Ca^2+^ required for their full proteolytic activation *in vitro*; the term μ-calpain reflects the concentrations of 1-100 μM Ca^2+^, whereas m-calpain 0,1-1 mM Ca^2+^ respectively (Ono and Sorimachi, 2012).

Calpains’ presence is reported in almost all eukaryotes and some bacteria, except for archaebacteria. They are proven to be expressed in all mammalian tissues with limited tissue-specificity. Only a minor fraction of calpain proteases become tissue-specific. Calpain III (historically known also as p94) can be used as an example of non-ubiquitous calpain, with its expression mainly restricted to the stomach (Sorimachi et al., 1993). Calpain-mediated proteolysis differs significantly from previously described proteolytic systems in a mode of action. Calpains act through proteolytic processing instead of completely degrading the protein. They perform somewhat limited hydrolysis of only one or a few sites in the substrate protein, modulating its structure and, ultimately, its activity or cellular function. For that reason, the calpain family is referred to as modulator proteases (Sorimachi et al., 2011). Still, the activity of calpain itself is tightly regulated by the other component, calpastatin, which represents the only endogenous specific inhibitor for conventional calpains.

The expression of calpains and calpastatin in human and murine lymphocytes is studied since the late 80’s of the XXth century (Takano et al., 1988; Murachi et al., 1990; Deshpande et al., 1993). Their function in the immune cells varies from regulation of protein kinase C (Shenoy and Brahmi, 1991), participation in activation and activation-induced cell death (AICD) of lymphocytes (Sarin et al., 1994; Deshpande et al., 1995), modification of T cell cytoskeleton and movement (Selliah et al., 1996; Stewart et al., 1998; Rock et al., 2000; Babich and Burkhardt, 2013), and intracellular signaling (Rock et al., 1997; Penna et al., 1999; Li and Iyengar, 2002; Hendry and John, 2004). Inhibition of calpains in activated peripheral blood mononuclear cells (PBMC) resulted in decreased secretion of IL-2 and reduced expression of CD25 on the surface of T cells (Schaecher et al., 2001). Its effect on intracellular signaling and migration of human monocytes was also documented (Noma et al., 2009). We have demonstrated that calpains are activated in stimulated human T cells (Mikosik et al., 2007), and that calpain inhibition in resting human T cells greatly reduces their proliferative response to polyclonal activation, secretion of multiple cytokines, as well as activation-induced phosphorylation of p56Lck, NFkB, and PLCgamma (Mikosik et al., 2016). Calpains and calpastatin constitute the calpain-calpastatin system (CCS), where both proteins are in stoichiometric balance in the cytoplasm and influence each other’s activity. We have shown this to be true for human T and B lymphocytes, simultaneously demonstrating that the amounts of both calpain 1 and 2 and calpastatin vary between CD4^+^ and CD8^+^ T cells and B lymphocytes (Mikosik et al., 2013). Like the aforementioned protein quality control systems, the calpain family is no exception to being physiologically relevant. It has been proven that calpains can regulate at least over 300 substrate proteins that are components of the signal transduction pathway, apoptosis, or immune system response (Ono and Sorimachi, 2012; Witkowski et al., 2021).

#### 2.2.4 Caspases

Caspases belong to the evolutionarily conserved family of cysteine-dependent endoproteases and hydrolyze their protein substrates sequence-specifically. When remaining inactive, caspases occur in the zymogen form. They undergo proximity-induced autoactivation triggered by dimerization-induced conformational changes where a linker between small and large subunits is proteolytically processed. Caspases are mainly responsible for inducing apoptosis, a homeostatic caspases process of programmed cell death, where either aged or damaged cells are eliminated. In apoptosis, caspases, depending on their function, fall into two distinct subtypes: initiator caspases (caspases 8, 9, and 10) or effector caspases (caspases 3, 6, and 7) that execute the ultimate cleavage of cellular components (Horvitz, 1999). Signaling events propagated by apoptotic caspases result in characteristic morphological changes in the cells undergoing apoptosis, and these typically include membrane blebbing, chromosomal DNA fragmentation, forming of apoptotic bodies, and cell death as the last step (Ramirez and Salvesen, 2018). What is more, an extensive body of evidence from past decades implicates caspases’ participation in another form of regulated cell death, pyroptosis, where pro-inflammatory caspases (such as caspase 1, caspase 5, and probably also caspase 4) execute cell termination. By definition, pyroptosis is a pro-inflammatory strategy for the regulated cell death and involves the secretion of the inflammatory cytokines interleukin (IL)-1β and IL-18 (Vande Walle and Lamkanfi, 2016). The innate immunity system is provided with pattern recognition receptors (PRRs) that scan the cellular environment for the presence of pathogen-associated molecular patterns (PAMPs) or host-derived molecules suggesting damage of host cells (DAMPs). Activated PRRs initiate an inflammasome assembly that subsequently recruits caspase 1 in the form of cytosolic zymogen to its active form. Later on, active inflammatory caspase 1 cleaves the inactive prointerleukins 1β and 18 to active IL-1β and IL-18 and gasdermin D (GSDMD) that oligomerizes to form membrane pores. It disrupts cell membrane integrity, which finally causes cell death and leakage of the pro-inflammatory cytokines and GSDMD itself (Liu and Lieberman, 2017). The important role of different caspases in T lymphocyte development and control of the immune reactivity (including proliferation, cell cycle progression, and the AICD/apoptosis) is well documented since decades (Denis et al., 1998; Los et al., 2001; Lakhani and Flavell, 2003; Schwerk and Schulze-Osthoff, 2003; Sehra and Dent, 2006; Brenner et al., 2008; Walsh and Edinger, 2010). Interestingly, it was recently shown that T lymphocytes, in addition to innate immune cells, harbor the components of classical and non-classical inflammasomes, which may get activated and, *via* various activation of caspase-1 and caspase 8, may be directed towards differentiated Th1, Th2 or Th17 and finally, similarly to innate immune cells, undergo pyroptosis upon GSDMD activation (Linder and Hornung, 2022).

#### 2.2.5 A disintegrin and metalloprotease and a disintegrin and metalloproteinase with thrombospondin motifs proteases

A disintegrin and metalloprotease (ADAM) and a disintegrin and metalloproteinase with thrombospondin motifs (ADAMTS) belong to zinc-dependent endopeptidases. They play an essential role in maintaining organ and tissue homeostasis. At the same time, they are responsible for the disruption of extracellular matrix proteins in disorders such as cancer or rheumatoid arthritis (RA). The ADAMs are multidomain transmembrane proteins, and their activity is implicated in proteolysis, cell proliferation, and adhesion (Mullooly et al., 2016). They also constitute major proteases associated with the shedding of transmembrane protein ectodomains. Here, a notable example is a soluble form of “anti-aging hormone,” Klotho, the ectodomain of which is shed (mainly from the surface of kidney cells with the ADAM10 and ADAM17 proteases). The reduced levels of soluble Klotho are associated with shorter life and certain aging-related diseases, including chronic kidney disease and vascular calcification leading to cardiovascular complications (Saar-Kovrov et al., 2021). The ADAMTSs are secreted and multidomain proteases and in control of the extracellular matrix structure. Unlike ADAMs, members of the ADAMTS family show a rather narrow substrate specificity (Kelwick et al., 2015). For instance, ADAMTS4 and ADAMTS5 can degrade aggrecan and therefore contribute to cartilage erosion (Rose et al., 2021). In human lymphocytes (Lambrecht et al., 2018), the ADAM proteases (mainly ADAM10 and ADAM17) are involved in their communication with structural cells by cleaving Notch, CD23, and CD44. ADAM10 and ADAM17 cleavage is necessary for activation, differentiation, and migration of helper T cells and their interaction with B lymphocytes, by affecting multiple target proteins, including CD40L, CD25, CD137, and CD154. In addition, ADAM17 reduces the expression of IFN-γ (Lambrecht et al., 2018; Sezin et al., 2022). On the other hand, the ADAMTS proteases were as yet not extensively studied in the human immune system and if, then only in pathological settings. Thus, their role in the development of atherosclerotic lesions is being suggested, *via* modulation of macrophage functions in arterial intima (Solanki et al., 2018). Specific ADAMTS protease, the ADAMTSL5 is considered an autoantigen in psoriasis (which however has nothing to do with its proteolytic activity; the same protein is apparently increasing the frequency of IL-17A and IFN-γ-expressing CD8^+^ T-cells, but only in psoriasis patients and not in healthy individuals. There are so far no mechanistic explanations for this phenomenon (Furue and Kadono, 2019).

## 3 Changes in main cellular proteolytic systems during aging

**FIGURE 1:**
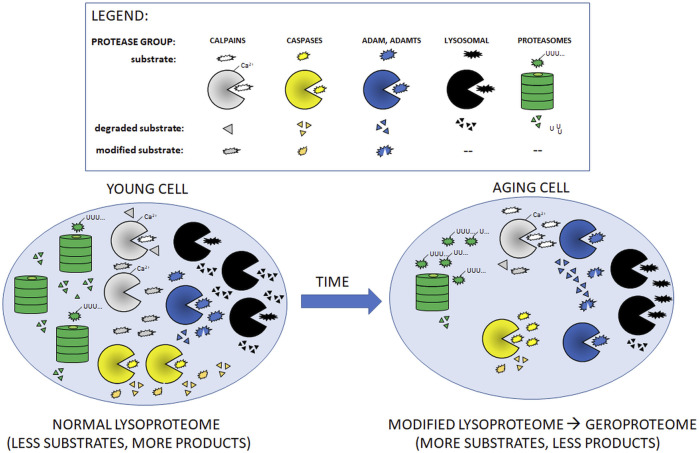
Cartoon presentation of aging-related changes in quantities/activities of main intracellular proteolytic systems described in Table 1. With the exception of ADAM and ADAMTS proteases, aging leads to decreased amounts/activities of the proteases (shown as fewer symbols of relevant protease in the aging cell as compared to young one). In consequence, aging cells accumulate more un-modified (non-functional) and undigested substrate proteins.

**FIGURE 2:**
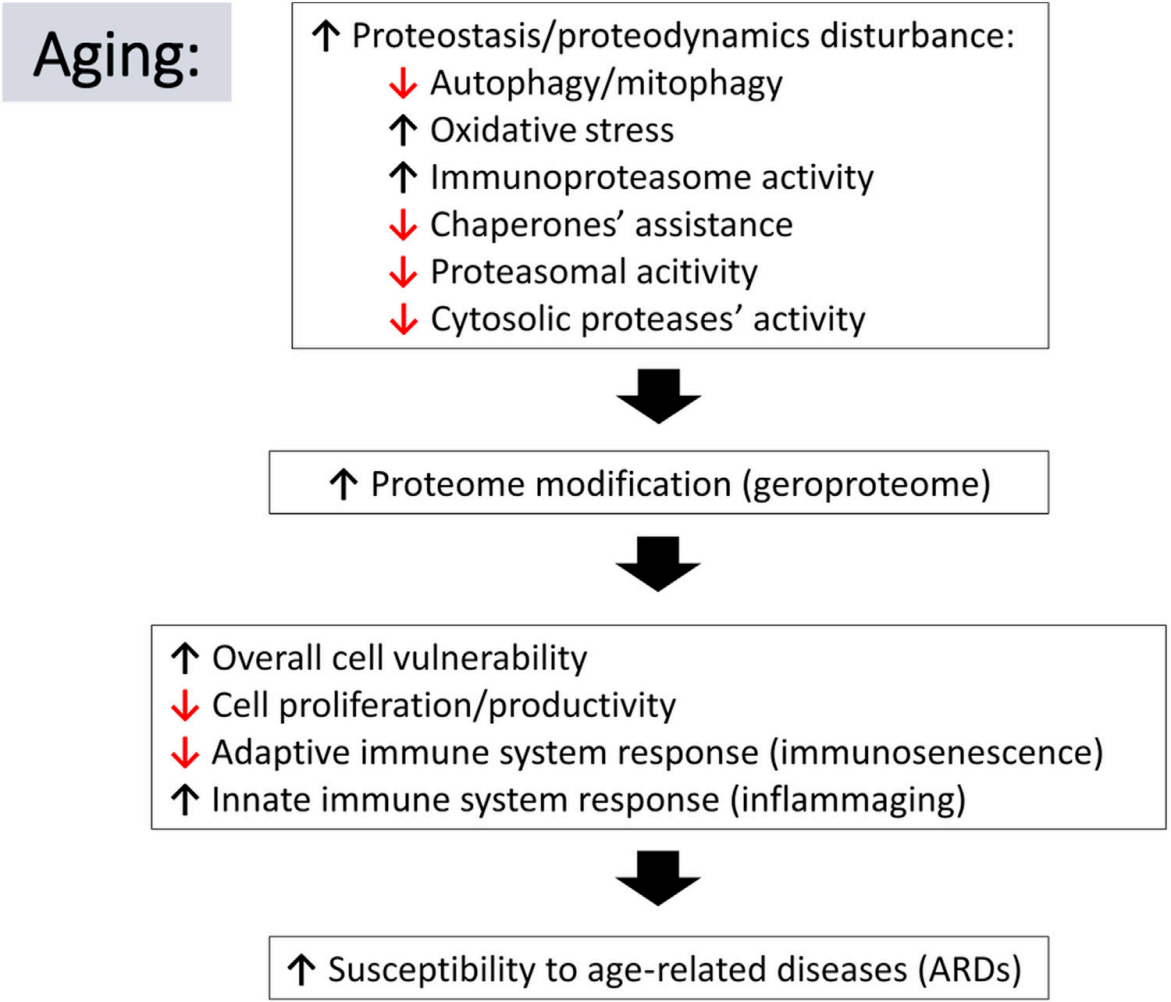
Schematic representation of relations between aging-associated changes in proteostasis/proteodynamics leading to modification of cellular proteomes and resulting in adverse consequences leading to the aging-related diseases. Arrow pointing down indicates a decline in the listed process, whereas an arrow pointing up suggests an increase.

An inevitable effect of aging is the loss of functionality of proteostasis network. It is well documented that the effectiveness of proteolytic systems decreases with age (Kaushik and Cuervo, 2015; Hipp et al., 2019). Cellular senescence is accompanied by accumulation of biological waste (including misfolded, aggregated, and otherwise impaired proteins and other molecules) and damage, primarily due to continuous exposure of a cell to oxidative stress (in this case, production of ROS) and increasing number of defective mitochondria (Terman et al., 2006) (Figures 1, 2). The accumulation of damaged and dysfunctional proteins due to impaired proteostasis and other molecules that cannot be effectively removed from the aging cell is termed “garb-aging” to indicate the role of this intracellular “garbage” in aging. Postmitotic cells (such as neurons and cardiac myocytes) that are long-lived, unable to undergo cell division, and distribute damaged molecules to daughter cells, belong to the biological entities especially affected by this garb-aging, as they have limited ways of getting rid of this intracellular “garbage” compared to the dividing cells (Franceschi et al., 2017; Tabibzadeh, 2021).

### 3.1 Changes within the ubiquitin-proteasome system

A vast literature supports the fact that proteasome activity during aging can be significantly decreased, which has been observed in a variety of aging cells or tissues, including lymphocytes (Carrard et al., 2003), spinal cord cells (Keller et al., 2000), fibroblasts (Chondrogianni et al., 2003), lens cells (Shang et al., 2001) or liver cells (Shibatani et al., 1996). There are different types of modification or regulation of proteasomes that could possibly contribute to its overall decline in activity with advancing cellular age. In one study, malfunctioning of proteasome originated from downregulation of catalytic subunits of the 20S proteasome complex and subunits of the 19S regulatory proteasome complex, leading to a decrease in the proportion of both 20S and 26S proteasome complexes (Chondrogianni et al., 2003). In another study, Ponnappan et al. (1999) discovered that induction of NFκB was severely compromised in the elderly compared to young donors. Examination of proteasome activity showed a significant decline in chymotryptic activity of the 26S proteasome, and the inability to degrade IκBα may imply the immune system dysfunction during aging. Another research group suggested that the rationale behind inefficient proteasome activity was uneven amounts of α- or β-subunits, thereby limiting the speed of catalytic proteasome formation or feasibly restricting the availability of α-subunits and β-subunits combinations to form multiple isoforms (Keller et al., 2000). Apart from that observation, faulty expression of regulatory subunits was also enlisted as a cause for lowered proteolytic activity (Ferrington et al., 2005). Divergent mechanisms accounting for protein quality system control dysfunction suggest that proteasome activity could be cell/tissue-type specific and heterogeneous. As mentioned previously, elevated age-dependent oxidation may impair proteasome activity. In order to irreversibly modify the proteolytic complex, the formation of 4-hydroxynonenal-adducts with particular subunits was performed in one study. In the case of the 20S proteasome, its chymotrypsin-like activity was promptly inhibited while interacting with oxidant 4-hydroxynonenal (Ferrington and Kapphahn, 2004). Moreover, the assembly of protein carbonyls within the regulatory subunit of the 26S proteasome, S6 ATPase, negatively affected the proteasome’s ability to degrade biomolecules (Ishii et al., 2005). Ubiquitin labeling of protein substrates seems to be affected by aging as well. While investigating bovine lens tissue a decline in levels of free ubiquitin has been documented, whereas during the gene expression profiling in age-related cataract transcription of an E3 ligase was downregulated (Hawse et al., 2004). Protein degradation by immunoproteasomes is affected by aging as well. Its subunits are also prone to modifications mentioned above.

Recently, decreased proteasome activities in aging lymphocytes upon their T-cell receptor (TCR) stimulation were shown to be associated with defective proliferation, cytokine production, and increased numbers of PD-1^+^CD44^high^CD4^+^ T cells which accumulate with aging. Altogether these findings suggest that reduced proteasomal activity underlies the deleterious process of immunosenescence (Arata et al., 2019). Decreased proteasomal activity in aging lymphocytes also results in less effective presentation of peptides for the MHC; pathogen-derived antigens may be elaborated improperly, and adaptive immune response may malfunction. Moreover, reduced proteasome-associated activity in the elderly was related to CD45RO^+^ and CD45RA^+^ T lymphocyte subsets that constitute naive and memory cells (Ponnappan, 2002). During maturation, differentiation, and aging of B lymphocyte lineage the amounts and total activities of proteasomes initially are high, but in differentiated plasma cell aging after the period of high secretion of immunoglobulins the abundance and in consequence, the activity of proteasomes is reduced this phenomenon is called the proteostenosis (Cenci et al., 2011).

### 3.2 Changes within the autophagy-lysosome pathway

It has been proven that macroautophagy and chaperone-mediated autophagy also belong to proteolytic systems undergoing the effects of aging, and their activity is therefore limited with age, similarly to that of the ubiquitin-proteasome system. For instance, extensive studies of the aging autophagy-lysosome system were performed on cardiac myocytes (Terman and Brunk, 2005), hepatocytes (Terman, 1995), retina cells (Rodríguez-Muela et al., 2013), (Carrard et al., 2003) or fibroblasts (Merker et al., 2000). In one of the earliest studies (Terman, 1995), results indicated that decreased autophagy in aging cells was due to faulty clearance of autophagic vacuoles. Although the organic content was efficiently sequestered by the double-membrane cargo vesicles in aged hepatocytes, the removal of autophagosomes by fusion with lysosomes was substantially hindered, and in consequence, degradation of the sequestered cargo was impaired. The intralysosomal, gradual accumulation of lipofuscin, mostly in postmitotic cells, also contributes to the failure of the lysosomal enzymes and, therefore, sluggish clearance of autophagosomes. Lipofuscin, autofluorescent, and yellowish pigment, is a highly oxidized cross-linked aggregate consisting mainly of protein and then lipid or carbohydrate material. Moreover, lipofuscin incorporates redox-active transition metals enabling producing even more reactive oxygen species *via* the Fenton reaction (Jung et al., 2007), and organelles become additionally susceptible to oxidative damage. Accumulation of lipofuscin was manifested biomarker of cellular aging, and its increasing rate was shown to be inversely related to life span (Jung et al., 2007). Another study pointed out that compromised endocrine regulation in aging may obey alterations in macroautophagy activity. In young organisms, when glucagon level increases during starvation, it results in upregulation of macroautophagy. In the case of insulin secretion, when nutrients are ingested, macroautophagy is repressed *via* the mTOR-mediated pathway. In aging organisms, the compromised stimulatory effect of glucagon may result from the basal signaling activity of the insulin receptor that becomes amplified by the oxidative stress even when insulin is lacking (Donati et al., 2008). A typical feature of aging cells is their possibility of developing gradual resistance to insulin as well. Such a scenario may deregulate the macroautophagy process and result in its continuous activation, leading to the depletion of necessary autophagy elements. Transcriptional downregulation of autophagy genes poses another alternative reason for age-related dysfunction in autophagy, for example, in the agent rodent muscle (Wohlgemuth et al., 2010). Expression of some autophagy-related genes, such as ATG5, was also found to be reduced (Rodríguez-Muela et al., 2013). In the case of chaperone-mediated autophagy, decreased level of LAMP-2A was also pointed out as an age-related consequence (Cuervo and Dice, 2000). Recently another aspect of reduced macroautophagy was studied in human T-cells, namely the impaired mitophagy (removal of damaged mitochondria). It was found that mitochondrial respiration, and consequently ATP production *via* the OXPHOS mechanism, is impaired in the mitochondria from aging CD4^+^ T-cells. These lymphocytes were burdened with numerous autophagosomes containing non-digested, presumably faulty, mitochondria. Defective mitophagy hampers mitochondria turnover and may trigger chronic inflammatory diseases and impaired adaptive immune defense in older individuals (Bektas et al., 2019). On the other hand, a very recent paper demonstrated that effective autophagy of mitochondria in memory B cells protects their survival and function (at least in mice) by reducing the oxidative stress; thus, impaired mitophagy may ultimately associate with decreased B cell reactivity to cognate antigens and production of antibodies (Vaena et al., 2021).

### 3.3 Aging of the calpain-calpastatin system

Even though calpains are involved in regulating the activity of at least 300 cellular proteins, the knowledge about these proteases in aging cells is very limited. In a study concerning the amounts of calpains and calpastatins in resting T lymphocytes (populations CD4^+^ and CD8^+^), B lymphocytes (CD19^+^), and monocytes, it was shown that amounts of calpain I and calpain II were significantly reduced in cells of healthy elderly in comparison to healthy young population (Mikosik et al., 2016; Witkowski et al., 2021). The assumption was made that the decrease in the amounts of calpains could partly contribute to the weakened immunity of the elderly to infections and cancers. What is more, the activities of calpain I and calpain II were remarkably reduced. As already mentioned, in our study, where the activity of calpains in resting T lymphocytes was inhibited, it led to a decrease in their response in proliferation to polyclonal stimulation *in vitro*, and production of multiple cytokines was lowered as well; the mechanistic cause of these effects was apparently reduction of amounts of phosphorylated p56lck, phospholipase C gamma, and NFκB (Mikosik et al., 2016). Our later observations have demonstrated that calpain activity may also affect the amounts of yet another molecule necessary for the T and B cell signaling, the phospho-ZAP70 (Łopatniuk et al., 2020). Results of these studies suggest that constitutive calpain activity plays an important role in the response of human immune cells to pathogens and, indirectly, that the reduced amounts and activities of calpains in the lymphocytes of aging human beings may be deleterious to their immune response efficiency.

### 3.4 Aging of the caspases

The process of regulated cell death also seems to be affected by aging in at least several cell types. In human adipose mesenchymal stem cells, expression of apoptosis genes was decreased with simultaneously increased expression of so-called senescence-related genes. There was a visible downregulation of genes encoding caspase 3, 8, and 9 (Alt et al., 2012). A similar scenario was seen in the studies of the murine bone marrow mesenchymal stem cells. Microarray analysis showed a decline in many apoptosis-associated genes’ expression, including caspase 8 (Wilson et al., 2010). A study concerning senescent fibroblasts in their final *in vitro* state of irreversible growth arrest showed that these cells were resistant to apoptosis. The study revealed that this resistance was associated with the lowered activity of caspase 3 (Marcotte et al., 2004). Executive caspase in the immune cells’ death by pyroptosis, caspase 1, and its role were investigated in atherosclerosis, a cardiovascular disease where advanced age is a risk factor. Research confirmed elevated levels of this caspase (Benjamin et al., 2017).

### 3.5 Aging of the a disintegrin and metalloprotease and a disintegrin and metalloproteinase with thrombospondin motifs proteases

When an organism is affected by environmental factors during the lifetime, it impacts the extracellular matrix remodeling process in aging cells, and its homeostasis is disturbed. Also, the regulation by ADAM and ADAMTS proteases is compromised and is associated with aging and age-related pathologies. For instance, a member of the ADAM family, ADAM17, is implicated in cancer formation and its progression. Higher levels of ADAM17 protease were found in multiple cancer types in comparison to the cellular environment of normal tissue (Mullooly et al., 2016). On the other hand, the role of ADAM17 in shedding the soluble ectodomain of Klotho (mentioned above) implies that lowered activity of this metalloprotease seen in the old cells may have multipronged effects on aging and related pathologies (Saar-Kovrov et al., 2021). Our group showed some time ago the reduced expression of cell-bound Klotho on the T cells of healthy elderly and acceleration of this reduction in rheumatoid arthritis patients (Witkowski et al., 2007). The mentioned ADAM10 and ADAM17 proteases were also shown to participate in shedding soluble CD137, a member of the TNF receptor family stimulating T cell proliferation, implicated in inflammatory diseases, autoimmunity, and cancer, i.e., diseases strongly associated with aging (Seidel et al., 2021). Also, ADAMTS genes were proposed to have an implication in cancer development. For instance, in human breast carcinoma, the expression of ADAMTS4 or ADAMTS6 was upregulated (Porter et al., 2004). Pro-inflammatory cytokines, characteristic for inflammaging that accompanies aging, influence ADAMs, and ADAMTs by increasing their production. Greater activity of these proteases is particularly visible in the pathogenesis of rheumatoid arthritis in inflamed synovial joints (Malemud, 2019). Finally, so far to our knowledge there are no published data directly implicating ADAM or ADAMTS proteases in the aging of human immune system cells including both lymphocytes and macrophages.

## 4 Role of age-dependent changes in cellular proteolytic systems for age-related diseases

Aging is characterized by an increased susceptibility to diseases and dysregulation of physiological processes. Many reports support the role of impaired protein quality control systems in the pathogenesis of age-related diseases (ARD), including chronic inflammatory diseases, neurodegenerative diseases, sarcopenia, atherosclerotic cardiovascular disease, and type 2 diabetes mellitus (T2DM) (Table 1) (Wong and Cuervo, 2010; Gouveia et al., 2017; Musci et al., 2018; Liao et al., 2021; Goh et al., 2022).

**TABLE 1:** Systemic summary of changes during aging in particular components taking part in proteolysis.

Proteolytic systems affected by aging	Effect of aging	Related disorder/pathology
Ubiquitin-proteasome system	• Deficiency in the chymotryptic activity of the proteasome	• Neurodegenerative diseases (e.g., Parkinson’s or Alzheimer’s disease, Huntington’s disease)
	• Disproportion in the 20S proteasome subunits	
	• Downregulation of catalytic subunits	• Weakened immune system response
	• Fewer amounts of free ubiquitin	
	• Oxidation associated adducts	
	• Perturbation of expression of regulatory subunits	
Autophagy-lysosome pathway	• Accumulation of lipofuscin	• Age-related macular degeneration
	• Downregulation of autophagy genes	
	• Faulty interference of the endocrine system	• Type 2 diabetes mellitus
	• Inefficient clearance of autophagic vacuoles	• Weakened immune system response
Calpain system	• A decline in amounts of calpain I and calpain II in T lymphocytes	• Weakened immune system response
Caspase system	• Downregulation of executive caspases	• Atherosclerosis
		• Cancer
ADAM and ADAMTS proteases	• Upregulation of ADAMs and ADAMTSs	• Cancer
		• Chronic kidney disease
		• Rheumatoid arthritis
		• Vascular calcification

Extensive research was done in the field of neurodegenerative disorders. Some of them belong to so-called protein-conformation disorders, including pathologies such as Parkinson’s or Alzheimer’s disease. A common feature of these disorders is either accumulation of autophagic vesicles or toxic protein aggregates that constitute ubiquitinated and improperly folded proteins. For example, the hallmark of Parkinson’s disease is the appearance of intracellular deposits of α-synuclein, a protein present at presynaptic nerve terminals (Takeda et al., 1998). In Alzheimer’s disease, β-amyloid protein is deposited outside neurons and in the vascular walls of the brain with simultaneous accumulation of hyperphosphorylated Tau-protein in the cell body of the neurons, dendrites, and axons during different stages (Duyckaerts et al., 2009). In metabolic disorders, including the T2DM, suppressed chaperone-mediated autophagy contributes to symptoms such as renal hypertrophy. Therefore, substrate proteins preferentially accumulate in the hypertrophic kidney along with reduced levels of hsc70 and LAMP-2A (Sooparb et al., 2004). When it comes to retinopathies, lipofuscin accumulated in the retinal pigment epithelium contributes significantly to age-related macular degeneration (AMD). The presence of A2-E, lipofuscin fluorophore, impairs degradation *via* lysosomes by inhibiting the ATP-driven proton pump (Bergmann et al., 2004).

The onset of old age manifests visible and characteristic changes that concern the functioning of the immune system. The main change is associated with increased levels of pro-inflammatory factors and related inflammatory processes leading to chronic inflammatory diseases. The inflammation status, which is low-grade and chronic, is defined as inflammaging and contributes to the imbalance of the immune system. For example, elevated levels of cytokines like IL-1, IL-6, and TNF-α are notably detectable during the inflammaging process in an aged population (Franceschi et al., 2000). Inflammaging during the aging of the immune system, together with proteostasis alterations, increases overall cell vulnerability. Inflammation was proposed to determine the rate of the aging process (Fulop et al., 2018). Still, clinical manifestations of age-related diseases are visible a long time after the disease onset. Age-related diseases are the effect of individual organism adaptations acquired during the whole lifetime (immunobiography), leading to concurrent immunosenescence and inflammaging (Fulop et al., 2020, 2021). With age, immunosurveillance of the organism falls. Therefore, the protective mechanisms against cancer are weakened, resulting in the progression of cancer (Fulop et al., 2013). Cardiovascular diseases are another example where protracted inflammaging can be seen as one of the causes of chronic age-related disease. In this case, atherosclerosis is also categorized as a chronic inflammatory process that manifests clinically (e.g., as coronary heart disease) after many years or even decades of its progression (Libby and Hansson, 2015). Atherosclerosis develops in the arterial wall in defined locations. Progressive changes may be initiated by pro-inflammatory cytokines, lipid unbalance, or past infections that could induce an inflammatory response. Last but not least is rheumatoid arthritis (RA), an autoimmune disease of still not fully understood etiology that primarily affects the joints but can attack other organs, including the nervous system, the heart, or the digestive system. RA’s pathology is strictly associated with a chronic inflammation process. Its incidence increases with age up to 80 years and is more prevalent among the female population (Radu and Bungau, 2021). It may be reasonably speculated that aging-associated failure of proteostasis in the cells of the immune system not only precipitates heir malfunction and related immunosenescence, but on the other side the accumulating, not degraded remnants of misfolded and aggregated proteins may trigger inflammatory readiness leading to inflammaging and then chronic inflammation of clinical dimension (Witkowski et al., 2021).

In the most recent theory of aging (oxi-inflamm-aging) (de Toda et al., 2021), the immune system is set as an example of the driver of aging that amplifies the oxidative-inflammatory stress that is associated with aging. It is widely accepted that oxidative stress contributes to the accumulation of damaged biomolecules and with aging the leakage of reactive oxygen species produced in mitochondria increases. Mitochondrial ROS react with mitochondrial DNA what finally results in mitochondrial dysfunction (Fuente and Miquel, 2009). Such a scenario additionally drives the increase in ROS production. When cells (both immune and non-immune cells) are exposed to excessive amounts of reactive oxygen species, in order to avoid malignant transformation, the cells enter senescence and therefore develop a senescence-associated secretory phenotype (SASP) (Coppé et al., 2010). Acquisition of SASP causes cells to secrete pro-inflammatory mediators in order to attract immune cells (e.g., macrophages, natural killer cells, and cytotoxic T cells) so they would imply tumor-suppressive mechanisms and eliminate non-functional or possibly malignant cells. During aging, homeostasis balance is disturbed and immune system functioning deteriorates (Feehan et al., 2021). It gives rise to chronic accumulation of senescent cells with simultaneously promoting inflammation what additionally enhances the response of immune cells and contributes to the generation of spiral feedback that includes inflammation and oxidation. All these actions ultimately lead to tissue/organ dysfunction and therefore accelerate aging.

## 5 Conclusion

During the inescapable aging process, any changes in the protein quality control systems, even these subtle ones, may disturb the fragile balance of proper functioning of the proteome. Impairment of protein turnover could result in faulty protein accumulation [conversion of the healthy proteome resulting from activities of multiple proteases (lysoproteome) to geroproteome (Witkowski et al., 2021) Figure 1], favoring pathophysiological processes and increasing the risk for chronic age-related diseases, particularly by affecting the function of the innate and adaptive immune system of the elderly (Figure 2).

## Abbreviations

ADAM, disintegrin and metalloprotease; ADAMTS, disintegrin and metalloprotease with trombospondin motifs; AMD, age-related macular degeneration; ARD, age-related diseases; ATG, autophagy-related genes; CCS, calpain-calpastatin system; CMA, chaperone-mediated autophagy; CR, caloric restriction; DAMPs, damage-associated molecular patterns; DRiPs, nascent oxidant-damaged proteins; GSDMD, gasdermin D; Hsc70, heat-shock cognate chaperone of 70kDa; HSFs, heat shock transcription factors; HSPs, heat shock proteins; IFNγ, interferon-gamma; LAMP2A, lysosome-associated membrane protein type 2A; MHC, major histocompatibility complex; mTOR, mechanistic target of rapamycin; MyoD, myogenic transcription factor HLH-1; PAMPs, pathogen-associated molecular patterns; PKK, phosphoinositide 3 kinase-related kinase; PN, proteostasis network; PPRs, pattern recognition receptors; RA, rheumatoid arthritis; ROS, reactive oxygen species; SASP, senescence-associated secretory phenotype; T2DM, type 2 diabetes mellitus; TNF-α, tumor necrosis factor-alpha; UPR, unfolded protein response; UPS, ubiquitin-proteasome system
